# Supplementary material for: Evaluation of Selected Plant Volatiles as Attractants for the Stick Tea Thrip Dendrothrips minowai in the Laboratory and Tea Plantation
Source: Insects. 2022 May 28;13(6):509. doi: 10.3390/insects13060509 (PMC9224518; doi:10.3390/insects13060509)
Supplement: Supplementary file 1 [file insects-13-00509-s001.zip › Supplementary-Table-S1.pdf]

Table S1. Related information of standard compounds.

Standard compounds	CAS	Purity（%）	Manufactory
4-Acetylpyridine	1122-54-9	97	Sigma-Aldrich
p-Anisaldehyde	123-11-5	98	Sigma-Aldrich
Decanal	112-31-2	98	Sigma-Aldrich
Eugenol	97-53-0	99	Sigma-Aldrich
Farnesene, mixture of isomers	/	/	Sigma-Aldrich
Geraniol	106-24-1	97	Sigma-Aldrich
(Z)-3-Hexenol	928-96-1	98	Sigma-Aldrich
(Z)-3-Hexenyl butyrate	16491-36-4	98	Sigma-Aldrich
Limonene	138-86-3	95	Sigma-Aldrich
Methyl anthranilate	134-20-3	99	Sigma-Aldrich
Methyl benzoate	93-58-3	99	Sigma-Aldrich
3-Methyl butanal	590-86-3	97	Sigma-Aldrich
Methyl isonicotinate	2971-79-1	98	Sigma-Aldrich
Methyl salicylate	119-36-8	99	Sigma-Aldrich
β-Myrcene	123-35-3	≥90	Sigma-Aldrich
Nonanal	124-19-6	98	Sigma-Aldrich
(E)-β-ocimene	13877-91-3	≥90	Sigma-Aldrich
(-)-α-Pinene	7785-26-4	99	Sigma-Aldrich
(+)-α-Pinene	7785-70-8	98	Sigma-Aldrich
γ-Terpinene	99-85-4	97	Sigma-Aldrich
