# Supplementary material for: Evaluation of Selected Plant Volatiles as Attractants for the Stick Tea Thrip Dendrothrips minowai in the Laboratory and Tea Plantation
Source: Insects. 2022 May 28;13(6):509. doi: 10.3390/insects13060509 (PMC9224518; doi:10.3390/insects13060509)

Supplementary Figure:

Figure S1. H-tube olfactometer for behavioral bioassay of *Dendrothrips minowai*.

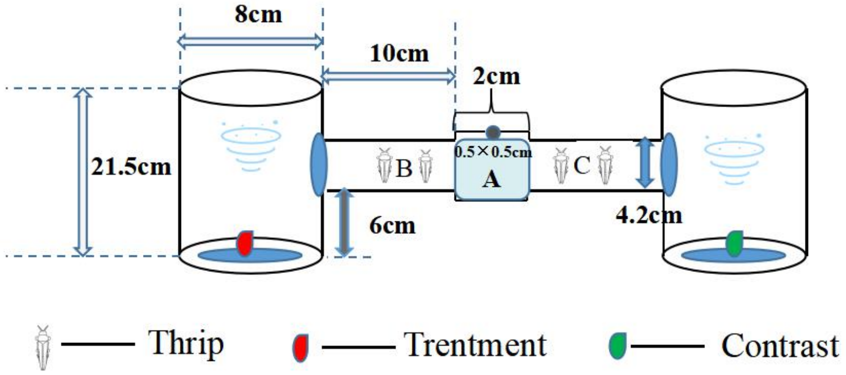

Supplement: Supplementary file 1 [file insects-13-00509-s001.zip › Supplementary-Figure-S1.pdf]
